# Exploiting Multiple Optimizers with Transfer Learning Techniques for the Identification of COVID-19 Patients

**DOI:** 10.1155/2020/8889412

**Published:** 2020-11-23

**Authors:** Zeming Fan, Mudasir Jamil, Muhammad Tariq Sadiq, Xiwei Huang, Xiaojun Yu

**Affiliations:** ^1^School of Automation, Northwestern Polytechnical University, Xi'an 710129, China; ^2^Ministry of Education, Key Laboratory of RF Circuits and Systems, Hangzhou Dianzi University, Hangzhou 310018, China

## Abstract

Due to the rapid spread of COVID-19 and its induced death worldwide, it is imperative to develop a reliable tool for the early detection of this disease. Chest X-ray is currently accepted to be one of the reliable means for such a detection purpose. However, most of the available methods utilize large training data, and there is a need for improvement in the detection accuracy due to the limited boundary segment of the acquired images for symptom identifications. In this study, a robust and efficient method based on transfer learning techniques is proposed to identify normal and COVID-19 patients by employing small training data. Transfer learning builds accurate models in a timesaving way. First, data augmentation was performed to help the network for memorization of image details. Next, five state-of-the-art transfer learning models, AlexNet, MobileNetv2, ShuffleNet, SqueezeNet, and Xception, with three optimizers, Adam, SGDM, and RMSProp, were implemented at various learning rates, 1e-4, 2e-4, 3e-4, and 4e-4, to reduce the probability of overfitting. All the experiments were performed on publicly available datasets with several analytical measurements attained after execution with a 10-fold cross-validation method. The results suggest that MobileNetv2 with Adam optimizer at a learning rate of 3e-4 provides an average accuracy, recall, precision, and *F*-score of 97%, 96.5%, 97.5%, and 97%, respectively, which are higher than those of all other combinations. The proposed method is competitive with the available literature, demonstrating that it could be used for the early detection of COVID-19 patients.

## 1. Introduction

Novel coronavirus, also known as COVID-19, emerged from the city of Wuhan, China, in late 2019. A disease that appeared to be like regular flu at first has now been officially declared as pandemic and has affected more than five million people so far around the world [1, 2]. Researchers believe that COVID-19 is a type of severe acute respiratory syndrome coronavirus 2 or SARS-CoV-2 [3]. Globally, coronavirus cases have crossed five million in numbers, with the death toll surpassing 320,000 [4]. The World Health Organization (WHO) declared a worldwide health emergency on Jan 30, 2020, to point out the alarming situation. Since then, most of the countries are under lockdown, with virus cases still inclining at a rapid rate.

The health system of many developed countries reached a point near collapse due to the pandemic [5]. Even with the latest medical facility available at hand, the only option that seems to be working against this disease is social distancing. Latest stats [6] have shown that China has efficiently defeated the virus through strict precautions and social distancing; however, the United States of America, Spain, Italy, France, and many other countries took a devastating hit from the virus. Doctors are using chest scans to diagnose the symptoms of COVID-19 rather than waiting for blood results [7]. Patients suffering from this disease have shown common signs like open holes in the lungs [8]. This research is being used to classify the patients and distinguish them from healthy people. Computed Tomography (CT) and chest X-ray provide huge assistance to doctors in diagnosing an infectious disease [9–11]. Obtaining scanned images through X-ray is faster, simpler, and economical as compared to CT scans [5]. In China, strict precautions were taken at the start to overcome the spread of this disease. Patients who showed mild level symptoms were quarantined and tested multiple times days apart to ensure the safety of other people [12].

Due to the severance of COVID-19, many researchers are proposing different methods to identify the infection through early symptoms and take the required measurements [13]. Even with so many available methods for the detection of the COVID-19 symptoms, there can still be a lack of affirmation due to false or inadequate results. Chest X-ray proves to be an excellent method, yet there is room for improvement in outcome accuracy. Computed tomography (CT) chest scans are processed through multiple stages to detect and narrow down the damaged region with the help of AI techniques [14, 15]. Some recent studies showed promising results in the early detection of COVID-19 signs. Narin et al. [12] have used three transfer learning models on the chest X-ray dataset to detect COVID-19 symptoms. They have achieved maximum accuracy in most of the folds, which can be an indication that the models may have shown overfit results. Wang et al. [16] fed CT images to a deep learning model, which can reveal damaged areas and extract required features that may help in diagnosing the disease. Shan et al. [17] used deep learning to develop a system that would automatically make multiple segments of lungs and reveal the infection.

Artificial intelligence and neural networks are being used readily in medicine to predict these kinds of viral diseases earlier through common symptoms [18]. Convolutional neural network (CNN), which is a type of deep learning, uses the images to train deep models and classifies them based on output categories [19]. This study is being used by researchers to train some state-of-the-art open-source neural network models and classify COVID-19 images. This branch of CNN is called transfer learning [20].

This paper suggests a deep learning approach to anticipate COVID-19 symptoms in a patient with the help of chest X-ray scans. In this study, transfer learning techniques were preferred over other machine learning algorithms due to the excellent classification accuracy of pretrained models, which also save time by avoiding the trouble of training and verifying the model weights from scratch. We have used five state-of-the-art predesigned networks in this study, including AlexNet, MobileNetv2, ShuffleNet, SqueezeNet, and Xception. These networks were fine-tuned by freezing most of the top convolutional layers and fully connected layers. Through multiple experiments on acquired datasets, we observed that the significant portion of the transfer learning model relies on the last convolutional layer for feature extraction and last fully connected layer for classification, which cannot be generalized for every dataset. Hence, only these layers were allowed to train weights. This approach not only saved a good amount of time but also provided competitive output accuracy. Moreover, each model is trained and tested on multiple optimizers as well as numerous learning rates to nullify the generalization factor, which is a crucial issue when there is a small amount of input data. The main highlights of the article are pointed as follows:

  (i)Five state-of-the-art transfer learning models are used with a fine-tuning approach to reduce training time while keeping the output accuracy intact
(ii) Each model is trained multiple times at different learning rates to reassure that the models are not overfitting and showing false results
(iii) MobileNetv2, when trained with the correct optimizer, provided the best results for chest X-ray images, although it is generally designed for mobile devices operations
(iv) The proposed method is effective and robust due to the verification of several statistical measures obtained with a 10-fold cross-validation approach

The rest of our paper is as follows: Section 2 describes the datasets used in this study, Section 3 explains our proposed methodology, Section 4 examines the analytics metrics, Section 5 exhibits the results against both datasets, Section 6 discusses and compares our study with previous research while Section 7 concludes the paper.

## 2. Materials

For this study, two datasets were used to validate the transfer learning models' efficiency on X-ray images. The first dataset [21] contains 74 “normal” and 74 “pneumonia” images for training. It was taken from GitHub. 20 “normal” and 20 “pneumonia” images were used to test the integrity of models. The same number of images was used for the second dataset, where “normal” scans were taken from [21], while infected “pneumonia” ones were acquired from another open source [22]. The datasets were acquired from public source collection. Datasets are being updated on a regular basis, so the number of collected images may differ in future studies. It is shown in Table 1 that different pretrained models take different input sizes. Hence, all images were resized according to each pretrained model requirement training.

## 3. Methodology

**Figure 1:**
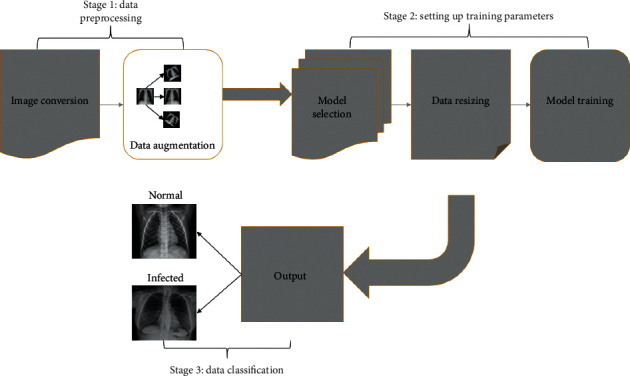
Block diagram of our study. Stage 1: data preprocessing: the number of channels of different images was made alike and data augmentation was performed. Stage 2: setting up training parameters: training parameters like number of epochs, mini-batch size, and number of folds per epoch opted in this stage. Data training was performed on each model after image resizing according to the distinct input size. Stage 3: data classification: here, our trained model displays the classified result as either normal or infected.

This paper suggests an approach to detect COVID-19 in patients via chest X-ray scans. The proposed method contains three stages. The first stage works on preprocessing that was done on the data after it was obtained from open-source collection. Multiple images in the dataset had a different number of channels, so they could not be processed in model training. Initially, all images were converted to the same number of channel, that is, 3 in our case. As our input data are not big enough, to ensure that we get good output results, different data augmentation techniques, including rotating, image flip, and pixel change, have been used. During the second stage, different training parameters like the number of epochs, optimizer selection, number of folds per epoch, mini-batch size, learning rate, and model were defined. Data were resized distinctly according to each model. The third stage performs the classification step in which the network decides whether there are COVID-19 symptoms in the scans. As detection of this disease is a sensitive case, numerous test runs were carried out to monitor the validity of trained models. Each transfer learning model was trained with three different optimizers, i.e., Adam, SGDM, RMSProp, and four learning rates, i.e., 1e-4, 2e-4, 3e-4, and 4e-4, to find the best combination and eliminate the factor of overfitting in data training. A flowchart of our suggested approach is shown in Figure 1.

### 3.1. Data Preprocessing

**Figure 2:**
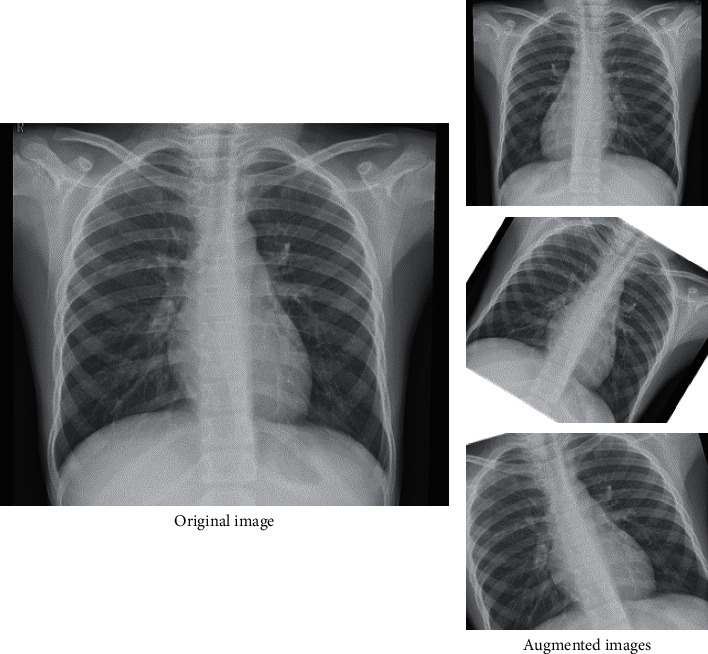
A visual representation of data augmentation.

Both datasets were processed through two stages to endorse the maximum output accuracy. Due to the presence of different numbers of channels for different images, all images were converted to the same channel size during the first stage. Neural network models require substantial data for training. As our input data was not large enough, data augmentation was performed to ensure that each model fed on enough input images to avoid overfitting. Data augmentation is a process in which images are modified by applying small changes in the original pictures like rotation, flipping the image, and minor adjustment in pixel range. An example of data augmentation is shown in Figure 2.

### 3.2. Transfer Learning

Deep learning focuses on functioning as a human mind. When a child is taught about different animals, an arbitrary image is formed in the mind of the child that a dog looks like this and a cat looks like this, and in the future, the child can recognize these animals. Deep learning works on the same principle. Transfer learning is the next step in deep learning. Training a neural network model requires a lot of time and multiple runs to capture the accurate weights according to the model's requirement. It is a tedious work and is not easy for students new to the field to enter transfer learning. Transfer learning handles the models shared by field experts for the public, which skips the requirement of finding compatible weights and carries on to the next step of the training model on new input data. We have used following pretrained models in our study:

- AlexNet
- MobileNetv2
- ShuffleNet
- (iv)SqueezeNet
- (v)Xception

**Figure 3:**
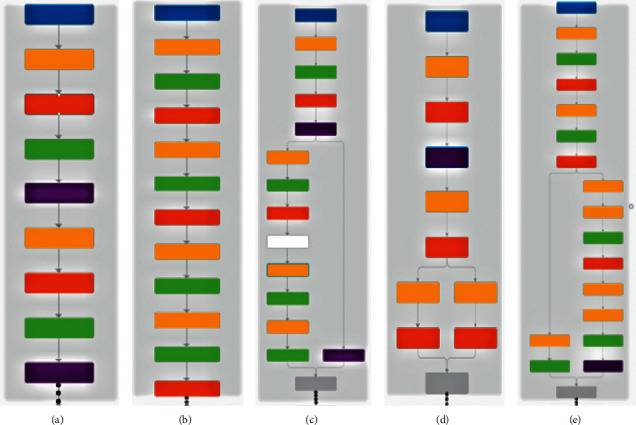
Frameworks of pretrained models: (a) AlexNet, (b) MobileNetv2, (c) ShuffleNet, (d) SqueezeNet, and (e) Xception.

Figure 3 gives visual on the architecture of these models. The blue block defines the input. Yellow indicates the convolutional layer. Orange box performs rectified linear unit (ReLU) operation. Green is responsible for cross channel normalization. The purple box is used for normalization, gray box concatenates the above results, and the white box represents channel shuffling. Some functions are unique to some models. For instance, MobileNetv2 executes clipped ReLU operation instead of general ReLu, and ShuffleNet contains multiple grouped convolutional layers.

#### 3.2.1. AlexNet

Given less number of computational parameters, as compared to other models with comparable performance for nearly every data form, AlexNet is one of the most famous pretrained models among researchers. It has five convolutional layers and three fully connected layers for classification purposes [23]. We only used the last convolution layer for feature extraction and the final fully connected layer for classification to reduce training time while keeping the accuracy unscathed. The image input size for AlexNet is 227 × 227 × 3.

#### 3.2.2. MobileNetv2

Originally designed for mobile devices by Google, MobileNetv2 is a fine pretrained model that delivers high output accuracy. It is designed to work with low input resources and reduced mathematical calculations. The working principle of this model is depthwise separable convolution and linear bottlenecks [24]. The second version of MobileNet, or as we call it MobileNetv2, also introduced short connections between bottlenecks. The input size of this model is 224 × 224 × 3.

#### 3.2.3. ShuffleNet

ShuffleNet is an extremely efficient model of a convolutional neural network that was also initially designed for mobile devices. It has an impressive computational power of 10–150 MFLOPs. This model operates on pointwise group convolution. ShuffleNet works on channel shuffling to reduce computational parameters and achieve high output accuracy. The model has proven work better than the “MobileNet” system for the classification of images [25]. It takes an input frame of the size 224 × 224 × 3.

#### 3.2.4. SqueezeNet

This 18-layer deep convolutional network was designed to achieve similar accuracy as AlexNet with 50x fewer parameters to compute. The idea behind reducing computation parameters is through the replacement of 3 × 3 filters with 1 × 1 filters. By performing this small operation, the model requires nine times calculations to perform. Another important concept of SqueezeNet is “Fire Module”. The squeezed layer feeds into the expand layer (3 × 3 filter) to reduce filter size and hence calculations. This architecture is known as “Fire Module” [26]. SqueezeNet has a similar input size as AlexNet.

#### 3.2.5. Xception

Xception or extreme version of the inception model is a pretrained neural network that operates on modified depthwise separable convolution. In simple depthwise convolution operation, channelwise nxn spatial convolution is being performed, while in the modified version (Xception), pointwise convolution is followed by depthwise convolution [27]. Xception has outperformed VGG, ResNet, and Inception-v3 in ImageNet competition. The input size of Xception is 299 × 299 × 3. Some key features of these models are presented in Table 1.

### 3.3. Matlab Application

**Figure 4:**
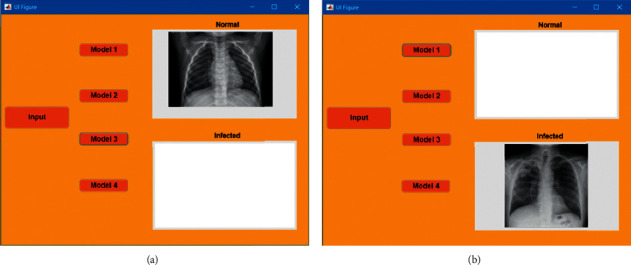
The interface of Matlab App is designed according to this study. (a) indicates when the model(s) classify the input image as to normal, while (b) symbolizes infected or pneumonia class.

An application related to our study was designed in the graphical user interface environment (Matlab GUI) of Matlab 2019b. It will assist researchers in the future study for coronavirus detection through chest X-ray images. Matlab app is a built-in program that is used to automate the required task. Multiple test runs were performed to corroborate the image ranking and run-time of the App. To get the best possible classification outcome, the finest transfer learning models used in this analysis, including “MobileNetv2” (against two different optimizers), “SqueezeNet”, and ‘Xception', were incorporated into the app. These networks have provided excellent classification results on chest X-ray images in our study.  Figure 4 portrays the app's function. It will take single image input and labeling the picture into one of our study-focused categories, i.e., normal or infected. Following are the components and their functions embedded in the app design:

1. Input: load the input data from the folder. Input will be in the form of an image; hence, the app is designed to accept file format of “.png”, “.jpg,” and “.jpeg”.
2. Model Selection: requires the user to select either of four models integrated into the framework of the app, where Model 1 and Model 2 represent MobileNetv2 (RMSProp, LR: 3e-4) and SqueezeNet (Adam, LR: 3e-4) trained with the dataset with 1 image while Model 3 and Model 4 demonstrate MobileNetv2 (Adam, LR: 3e-4) and Xception (RMSProp, LR: 3e-4) trained with the dataset with 2 images.
3. Axes: display the classified image, i.e., if the models detect no symptoms of COVID-19, the image will be displayed in the “normal” axis; otherwise, it will be portrayed in “infected” axis.

Parameters like epochs, optimizer, learning rate, and mini-batch size are preselected in the models as it is required to use integrated models in the app.

## 4. Performance Measures

We have used a built-in Matlab deep learning toolbox to train different transfer learning models on our input data. Each model was trained using the 10-fold procedure to guarantee the validity of the result. Each training run consisted of 10 epochs and 140 iterations per epoch. Models were fine-tuned before training. All the layers except the last one were frozen to avoid extra time consumption. The classification layer and the final fully connected layer of each model were replaced as they were originally designed to provide an output of 1000 distinctive categories. In each fold, out of 74 images, 15 images were separated randomly for validation tests.

All the training and test simulations were performed on an Intel Core i7-9750H processor enforced with 32 gigabytes of RAM and the GPU (graphics processing unit) Nvidia GeForce GTX 1660 Ti with 6 GB memory. Matlab framework was restarted before each new training to assure there is no false time consumption, which can incur when an excessive number of intense simulations are executed.

Detection of coronavirus among healthy people has become one of the top priorities of doctors worldwide. Results generated through these methods must be validated via multiple techniques because any false result can be very dangerous not only to that patient but also to other people in contact with that patient. All models were validated through analytics metrics, including overall accuracy, precision, recall, and *F*-score. The following equations represent the mathematical formulas of these metrics:

(1) $$\begin{array}{c} \text{Accuracy}=\;\frac{\text{TP}+\text{TN}}{\text{TP}+\text{TN}+\text{FP}+\text{FN}},\\ \text{Recall}=\frac{\text{TP}}{\text{TP}+\text{FN}},\\ \text{Precision}=\;\frac{\text{TP}}{TP+FP},\\ F-\text{measure}=\;\frac{2*\text{precision}*\text{recall}}{\text{precision}+\text{recall}}, \end{array}$$

where TP is “true positive”, TN is “true negative”, FP is “false positive,” and FN is “false negative,” respectively. These parameters are used to analyze the integrity of test results [28, 29]. Accuracy is the measurement of correctly classified samples in percentage or closeness of the measured value to a standard or true value. The number of positive class predictions from all positive examples in the dataset is defined as recall. Precision is the ratio of positive observations correctly predicted to the overall positive observations predicted, while *F*-measure gives a mean for both precision and recall to be integrated into a single measure that captures both properties. It is the harmonic mean of precision and recall.

## 5. Results

This study was carried out to diagnose patients with COVID-19 symptoms with the help of chest X-ray scans. Various deep learning models were trained and tested on multiple optimizers and several learning rates. The reason for performing this study on numerous parameters is to find the optimum combination of model, optimizer, and learning rate for our input data.

### 5.1. Dataset 1

**Figure 5:**
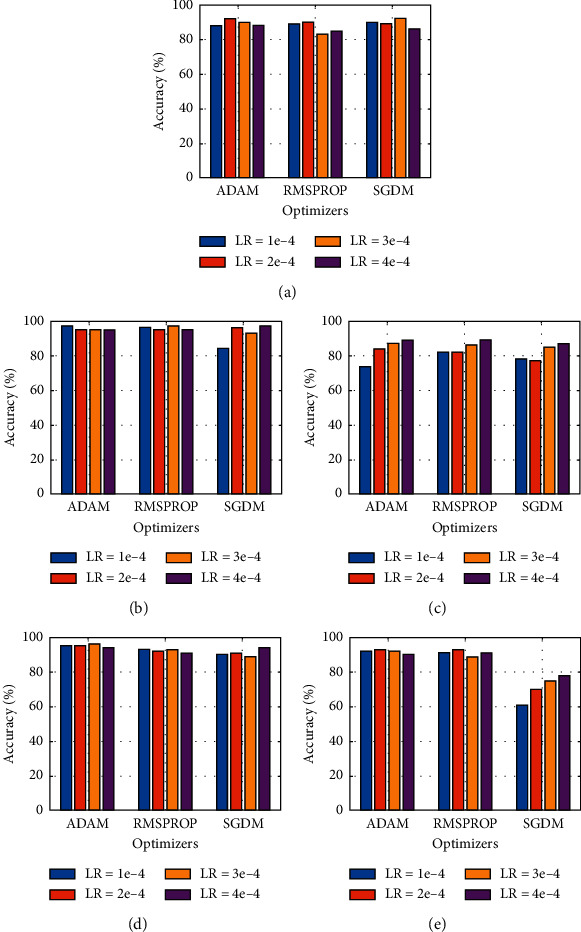
Graphical comparison of different models based on output accuracy for dataset 1: (a) AlexNet, (b) MobileNetv2, (c) ShuffleNet, (d) SqueezeNet, and (e) Xception.

Accuracy comparison of the first dataset is shown in Figure 5. We can see that MobileNetv2 adopted all three optimizers very well for all learning rates except for 1e-4, which is not uncommon in other models. MobileNetv2 synthesized the highest accuracy of 97% with “Adam” optimizer at a learning rate of 3e-4. ShuffleNet showed mixed results with a maximum output efficiency of 89% on two different combinations. For SGDM optimizer, Xception has shown surprisingly bad results falling up to 60% of average accuracy with LR = 1e-4, which is worse than all other scenarios in our study. SqueezeNet showed prominent results for all learning rates against different optimizers, reaching a maximum of 96% classification accuracy for Adam optimizer when the learning rate was selected as 3e-4.

**Figure 6:**
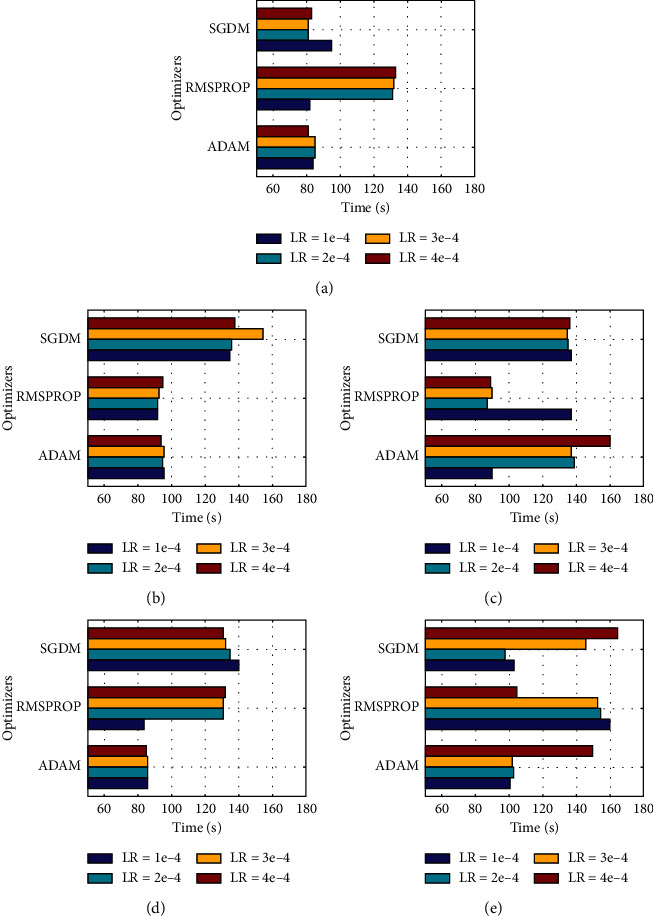
Time comparisons of all models for dataset 1: (a) AlexNet, (b) MobileNetv2, (c) ShuffleNet, (d) SqueezeNet, and (e) Xception.

Figure 6 depicts the training time of these models in different cases. On average, AlexNet has taken the least amount of time for each training except when trained with RMSPROP. MobileNetv2 expressed diverse training time for different optimizers. ShuffleNet and SqueezeNet both registered the maximum amount of computational time for all three optimizers, nearly approaching 100 seconds per run. AlexNet only consists of 8 layers, which is far less as compared to that of the other four models. So, its less time consumption is understandable, but MobileNetv2 results were somewhat surprising, taking far less training time and showing excellent classification results. Xception, as expected, required maximum training time as it is one of the most in-depth networks used in our study.

All in all, almost every model has adopted well with SGDM according to time usage. If we compare Figure 4 and Figure 5, we can quickly notice that SqueezeNet with Adam optimizer is probably the best combination of both accuracy and time consumption.

**Figure 7:**
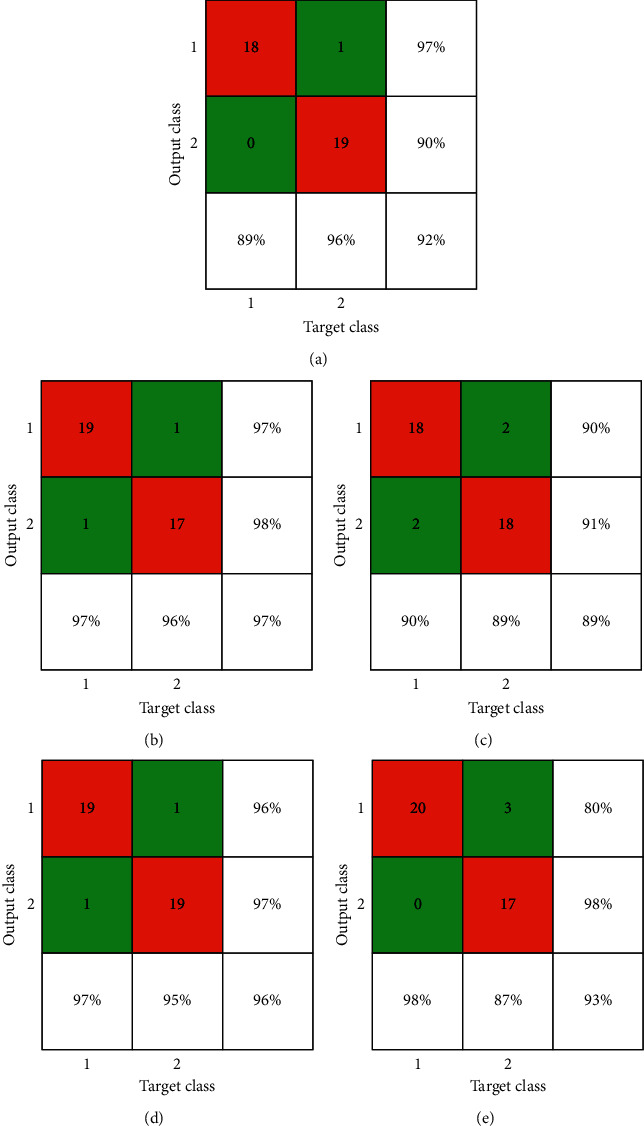
Confusion matrices of experimented models for dataset 1: (a) AlexNet, (b) MobileNetv2, (c) ShuffleNet, (d) SqueezeNet, and (e) Xception.

Confusion matrices for dataset 1 are shown in Figure 7, while Table 2 exhibits a comparison of precision, recall, and *F*-score for dataset: 1, where MobileNetv2 has attained the best F-score for both “normal” and “infected” classes. Again, “infected” here represent patients who showed pneumonia signs during medical tests. MobileNetv2 also got the highest precision score of 98% for “infected”, which is on par with Xception for the same case.

### 5.2. Dataset 2

**Figure 8:**
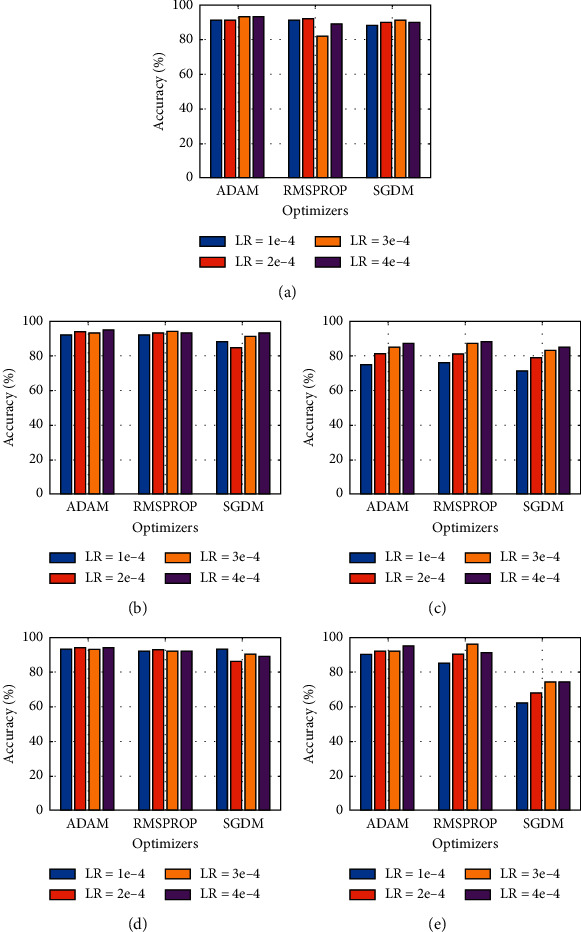
Graphical comparison of different models based on the output accuracy for dataset 2: (a) AlexNet, (b) MobileNetv2, (c) ShuffleNet, (d) SqueezeNet, and (e) Xception.

As mentioned before, we have used two datasets to verify the integrity of models. The following data re half part of the first dataset and the other half is extracted from another source [22]. Exact operations were performed on dataset:2 as were on dataset 1. A comparison of average accuracy for different models is given in Figure 8. Xception has shown a similar pattern here with SGDM. So, it is not recommended to use Xception for this dataset classification with either optimizer. Results can be improved with a big dataset as the Xception model works best on substantial data size, that is, if you want to use SGDM optimizer with Xception. However, a maximum result of 96% with RMSProp at LR: 3e-4 is still acquired, the best classification accuracy for dataset 2. An inclined configuration can be seen for MobileNetv2 when used with SGDM where output accuracy showed a direct relation with the learning rate, and it peaked at 95%.

**Figure 9:**
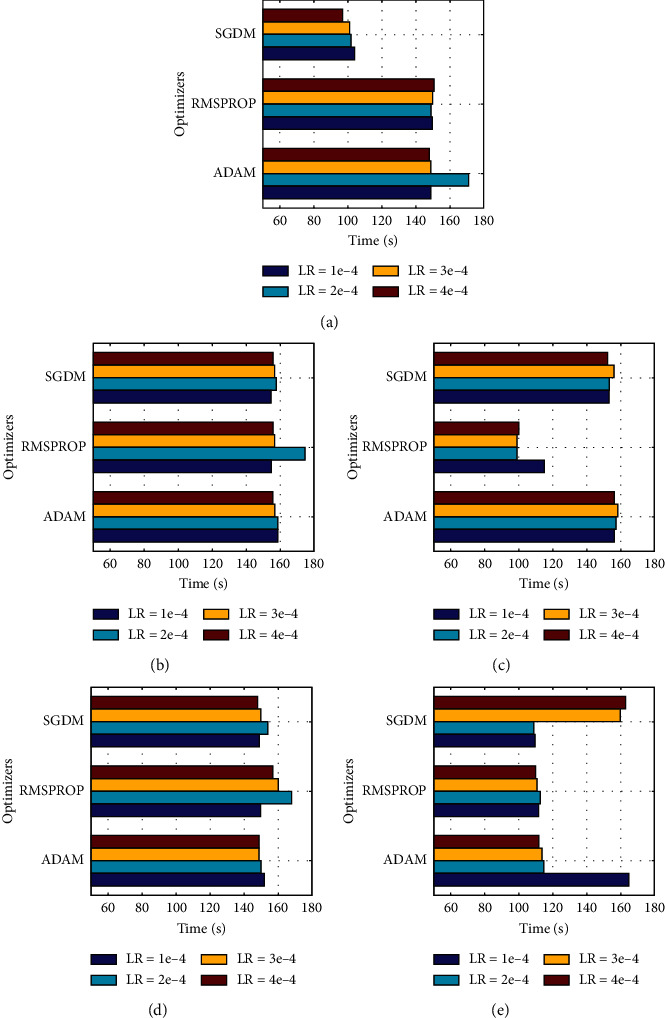
Time comparisons of all models for dataset 2: (a) AlexNet, (b) MobileNetv2, (c) ShuffleNet, (d) SqueezeNet, and (e) Xception.

However, with the other two optimizers, the results are very good for MobileNetv2. SqueezeNet has also produced excellent results with Adam as well as with RMSProp marking up to 94% output accuracy. The time consumption graph for each model against dataset 2 is shown in Figure 9.

Though Xception took the least amount of training time with this dataset, it is not recommended to use due to significantly less output accuracy. Other models show more or less similar results with little fluctuation where time is taken into account.

**Figure 10:**
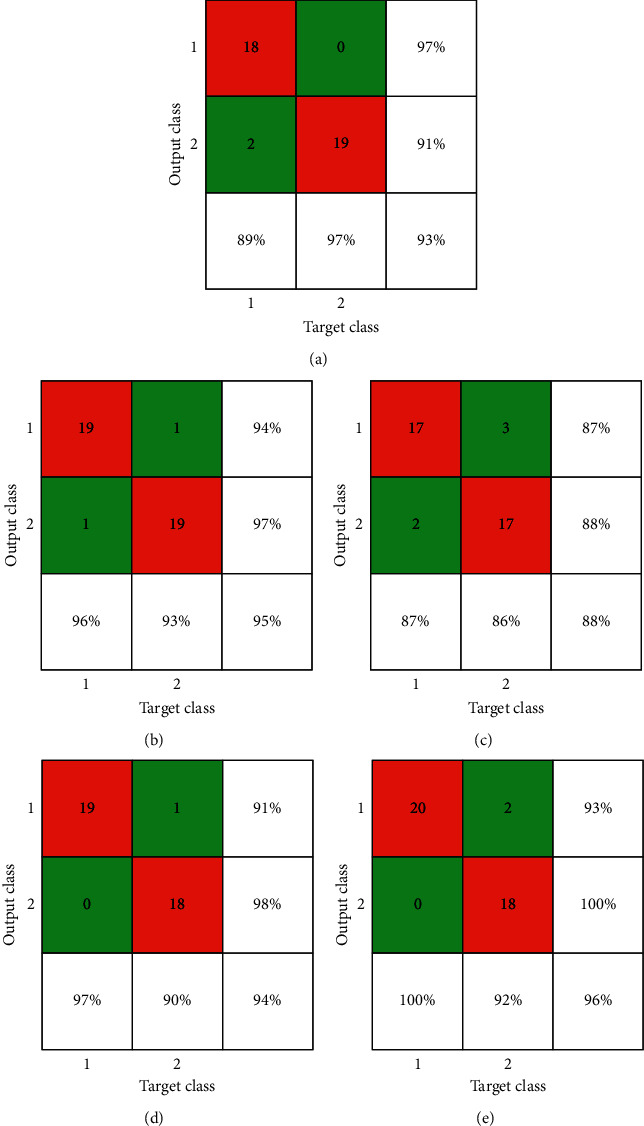
Confusion matrices of experimented models for dataset 2: (a) AlexNet, (b) MobileNetv2, (c) ShuffleNet, (d) SqueezeNet, and (e) Xception.

Figure 10 represents the confusion matrices of all models used in this study for the second dataset. Remarkably, Xception synthesized 96% accuracy as the best one; still, it did not show good average result. MobileNetv2 was second-best, which yielded 95% output accuracy.

Table 3 indicates that Xception attained the finest result with “normal” class while calculating recall, and it also measures 100% precision for “infected” class. That is why the F-score of Xception was the best among all the models. Table 3 and Figure 8 represent the best result that we observed. If we talk about average output, MobileNetv2 seems to be the clear winner.

## 6. Discussions

Numerous studies have been performed on the detection of COVID-19 symptoms via different techniques. Shan et al. [17] used VB-net for the image segmentations of patients. A study similar to ours was conducted in [12] where they achieved 98% accuracy. But, their results could be prone to overfitting as they did not use multiple optimizers or different learning rates and only used three transfer learning methods. Zhang et al. [30] performed X-ray images classification with the help of ResNet. Wang and Wong [31] adopted a convolutional neural network method for the classification of X-ray images. They successfully achieved 83.5% accuracy. A very famous transfer learning model “inception” was used by Wang et al. [16] to predict COVID-19 symptoms in CT images. The majority of these studies have performed classification on fewer neural network models as compared to our research. Furthermore, we have conducted training on different optimizers as well as on different learning to confirm that there is no overfitting going on due to the lack of big datasets. Our method is rigorous and repetitive, as we have performed 10-fold cross-validation.  Table 4 provides a quick overview of our findings as compared to several other studies that used similar datasets for neural network model training. Also, after carrying out numerous additional simulations, we have achieved near-best precision to ensure that the results produced are not false or due to a computational error. Our analysis is highlighted in the following points:

  (i)Multiple transfer learning models, including AlexNet, MobileNetv2, ShuffleNet, SqueezeNet, and Xception, have been used to classify chest X-ray images with different optimizers and learning rates to synthesize accurate results
  (ii)Fine-tuning has been used to reduce the computational parameters and make use of only those layers which take part in feature extraction
(iii) We have used X-ray images, which are not difficult to acquire, and showed that they could be beneficial in the detection of COVID-19 in a patient
  (iv)The problem with limited input data has been solved by making use of different data augmentation techniques

This study was just one way to diagnose COVID-19 symptoms in patients. Several other transfer learning models can be beneficial in image classification. Moreover, deep learning always relies on the amount of input data. Hence, if a large amount of data can be collected, it will further assist in getting enhanced results. For instance, Xception with SGDM showed relatively poor results due to insufficient input data.

## 7. Conclusion

Because of its fast-spreading potential, COVID-19 has rapidly become the key target of doctors and medical researchers around the world. It is critical to detect this virus in humans in the absence of a functional vaccine to prevent its dissemination. This paper emphasizes on using chest X-ray scans to diagnose COVID-19 symptoms. The proposed study implements five different transfer learning models with different optimizers and various learning rates on two public datasets. Results dictate that MobileNetv2 and Xception models can be instrumental in diagnosing coronavirus through chest X-ray images. To authenticate the effectiveness and robustness of trained model, all models were validated by several statistical indexes, including a 10-fold cross-validation method. We believe that this study can be a big help in the early detection of COVID-19.

## Figures and Tables

**Table 1:** Key features of the models used in this study.

Model	Input size	Number of layers	Parameters (millions)	Size (MB)
AlexNet	227 × 227 × 3	8	61.0	227
MobileNetv2	224 × 224 × 3	53	3.5	13
ShuffleNet	224 × 224 × 3	50	1.4	6.3
SqueezeNet	227 × 227 × 3	18	1.24	4.6
Xception	299 × 299 × 3	71	22.9	85

**Table 2:** Statistical measurement comparison of observed models for dataset 1.

Network	Class	Recall	Precision	*F*-score
AlexNet	Normal	89	96	92
	Infected	96	90	93
MobileNetv2	Normal	97	97	97
	Infected	96	98	97
ShuffleNet	Normal	90	90	90
	Infected	89	91	90
SqueezeNet	Normal	97	96	96
	Infected	95	97	96
Xception	Normal	98	89	93
	Infected	87	98	92

**Table 3:** Statistical measurement comparison of observed models for dataset 2.

Network	Class	Recall	Precision	*F*-score
AlexNet	Normal	89	97	93
	Infected	97	91	94
MobileNetv2	Normal	96	94	95
	Infected	93	97	95
ShuffleNet	Normal	87	87	93
	Infected	86	88	92
SqueezeNet	Normal	97	91	94
	Infected	90	98	94
Xception	Normal	100	93	96
	Infected	92	100	96

**Table 4:** Comparison of different studies.

Paper	Dataset	Objective	Approach	Highest avg. accuracy
Ghoshal et al. [32]	X-ray	COVID-19-image classification	CNN	92.9%
Pan et al. [8]	X-ray	COVID-19-image classification	ResNet50	98.0%
Zhang et al. [30]	X-ray	COVID-19-image classification	ResNet	95.18% (AUC)
Wang et al. [16]	X-ray	COVID-19-image classification	CNN	83.5%
Our paper	X-ray	COVID-19-image classification	MobileNetv2	97.0%

## Data Availability

The data used to support the findings of this study are available upon request by contacting the corresponding author.
